# Safe System Speed Management 

**Published:** 2019-08

**Authors:** Lori Mooren

**Affiliations:** ^*a*^Consultant with Safety and Communications Pty Ltd, Australia.

## Abstract

Speed management is crucial to a safe road and traffic system. Unsafe speeds travelled by motor vehicles contributes to around 40% of road fatalities. This lecture discusses the pivotal role of speed management in safe system road safety and how to address the problem of speeding.

With the adoption of Safe System principles for road safety strategies, it is no longer acceptable that people should die or be seriously injured due to mistakes made when using roads. The design and management parameter for road and traffic systems should focus on the fallibility and vulnerability of human road users. Speed is a most critical factor because it is the crash impact speed that determines the degree of harm that is inflicted on people.

Speed management is perhaps the most contested issue in road safety and the most difficult one to perform effectively. Part of this problem has to do with the very nature of human beings. Instead of making every effort to keep safe, people tend to always try to optimise the benefits of their risk behaviour. To make things worse, behavioural research has found that human beings do not generally judge risk accurately and have an optimism bias. So, when it comes to driving speed selection people tend to misjudge the time it will take for them to take evasive action or stop in time to avoid a crash. Perception, reaction and braking times will be covered in this lecture.

From crash data analysis and biomechanical research findings the impact crash speeds that can kill people in various crash types are now known. For example, the percentage of risk of fatally injuring a pedestrian rises sharply after the impact speed exceeds 30 km/h.

Road networks generally have a variety of functions. There are roads that have primarily flow through functions, roads with access functions and roads that serve to cater for a mix of vulnerable and less vulnerable road users. The Dutch first fostered the notion that road hierarchies should be defined, encompassing all of the functions that the road network aims to support. Then possible crash or traffic conflict types can be analysed.

If the overriding priority is to prevent road deaths, the safe limit for roads with possible conflicts between cars and unprotected road users is 30 km/h or lower. For locations where impacts with fixed roadside objects are possible the limit should be no higher than 40km/h. For intersections with possible lateral conflicts between cars 50km/h would be the maximum limit. Roads with possible head-on crashes between cars would have a limit no greater than 70 km/h. Where head-on and side-on conflicts with other road users are impossible, limits greater than 70 km/h can be considered. Motorways with side barriers and centre barriers should be limited to 110 km/h maximum. This lecture will discuss the actions that can be taken to prevent speed-related road injuries and fatalities. It will cover social, behavioural and engineering solutions.

**Keywords:**

Speed Management, Safe System, Road Safety

